# Microhardness of demineralized enamel following home bleaching and laser-assisted in office bleaching

**DOI:** 10.4317/jced.51705

**Published:** 2015-07-01

**Authors:** Majid Ghanbarzadeh, Farzaneh Ahrari, Majid Akbari, Haniye Hamzei

**Affiliations:** 1 DDS MS, Assistant Professor of Orthodontics, Dental Research Center, School of Dentistry, Mashhad University of Medical Sciences, Mashhad, Iran; 2 DDS MS, Associate Professor of Operative Dentistry, Dental Research Center, School of Dentistry, Mashhad University of Medical Sciences, Mashhad, Iran; 3 DDS, School of Dentistry, Mashhad University of Medical Sciences, Mashhad, Iran

## Abstract

**Background:**

There is little data regarding the effect of tooth whitening on microhardness of white spot lesions. This study was conducted to investigate the effect of home-bleaching and laser-assisted in-office bleaching on microhardness of demineralized enamel.

**Material and Methods:**

Forty bovine incisors were selected and immersed in a demineralizing solution for 12 weeks to induce white spot lesions. Enamel blocks were prepared and randomly assigned to two groups of 20 each. The first group underwent home bleaching with 15% carbamide peroxide which was applied for 8 hours a day over a period of 15 days. In the second group, in-office bleaching was performed by 40% hydrogen peroxide and powered by irradiation from an 810 nm gallium-aluminum-arsenide (GaAlAs) diode laser (CW, 2W). This process was performed for 3 sessions every seven days, in 15 days. The specimens were stored in Fusayama Meyer artificial saliva during the experiment. Surface microhardness was assessed before and after the bleaching therapies in both groups.

**Results:**

Microhardness decreased significantly following both home bleaching and laser-assisted in-office bleaching (*p*<0.05). There were no significant differences in hardness values among the two groups either before (*p*=0.131) or after (*p*=0.182) the bleaching procedures.

**Conclusions:**

Tooth whitening through home bleaching or laser-assisted in-office bleaching can result in a significant reduction in microhardness of white spot lesions. Therefore, it is suggested to take protective measures on bleached demineralized enamel.

** Key words:**White spot lesion, bleaching, laser, microhardness, demineralized enamel, home bleaching, in-office bleaching.

## Introduction

Bleaching is one of the most popular esthetic procedures in modern dentistry, aiming to give patients a more perfect appearance and esthetic smile. Vital teeth are usually whitened by one of the two techniques including in-office bleaching and night-guard or home bleaching. In-office bleaching employs relatively high concentrations of bleaching agents for short durations, whereas in home bleaching procedure, lower concentrated bleaching gels are applied over a longer period of time for example 4-8 hours a day for 2 or more weeks. Although the use of home bleaching technique is remarkably increased in recent years, the long period of treatment, the discomfort from wearing the trays, the unpleasant taste of the bleaching gel and the lack of compliance in some patients encouraged the clinicians to continue in-office bleaching (1) and even use supplementary modalities to enhance the whitening process. In-office bleaching is also preferred in cases of severe discoloration in one or more teeth (2), as the treatment can be localized in the target area.

The in-office bleaching process can be accelerated by the application of high-intensity light sources such as halogen curing lamps, plasma arc lamps, light-emitting diodes (LEDs) or lasers (2). Different wavelengths of lasers have been employed in recent years to enhance the whitening process through photochemical or photothermal interactions. The gallium-aluminum-arsenide (GaAlAs) diode laser is effective for a variety of dental applications including gingivectomy, fiberotomy, soft tissue removal and reducing tooth hypersensitivity, and it can also be employed for in-office bleaching (3,4).

Enamel demineralization or formation of white spot lesions is frequently observed in patients with high risk of caries such as those undergoing fixed orthodontic therapy (5,6). Tooth whitening has been considered as a treatment measure for improving the esthetic appearance of white spot lesions (7,8) unresponsive to remineralization treatments. A great number of patients undergoing orthodontic therapy are affected with extensive discolorations around their attachments for which tooth whitening may be recommended at the end of the treatment. Regarding the high incidence of white spot lesions in these subjects, bleaching can be employed to not only eliminate the discolorations, but also to improve the appearance of the dentition by masking the demineralized enamel (7,8), thus enhancing the esthetic outcome of orthodontic therapy.

Tooth whitening process includes direct contact of the bleaching gels on the tooth surface for a long period of time. This has induced concerns regarding the potentially adverse effects of bleaching agents on tooth structure (9,10). There are extensive though conflicting data regarding the effects of bleaching therapies on mechanical properties, micromorphology and chemical composition of enamel (11). However, few studies evaluated the effect of tooth whitening on microhardness of white spot lesions. As the mineral content of enamel is reduced in white spot areas, it is possible that the bleaching procedures produce more detrimental effects on demineralized than normal enamel. There are differences between home bleaching and in-office bleaching regarding the concentration and duration of application of the bleaching agents, and thus the impact of these procedures on mechanical properties of demineralized enamel might be different. This study aimed to examine the effect of home-bleaching and laser-assisted in-office bleaching on microhardness of enamel white spot lesions.

## Material and Methods

-Preparation of the specimens

Forty freshly extracted bovine incisors without caries, cracks or other enamel anomalies were selected and stored in distilled water at room temperature until the time of the experiment. The teeth were cleaned with water slurry of pumice and brush and then immersed in a cariogenic solution for 12 weeks to induce white spot lesions. The cariogenic solution consisted of 2.2 mM CaCl2, 2.2 mM NaH2PO4 and 50 mM acetic acid with pH adjusted at 4.8 using potassium hydroxide (KOH) (12). This solution was changed weekly. The teeth were then randomly assigned to two groups of 20 each. One group underwent home bleaching and the other one was submitted to laser-assisted in-office bleaching.

-Microhardness assessment

Before bleaching, the teeth were subjected to microhardness measurement. For this purpose, the crowns were sectioned from the roots and then were fixed in epoxy resin, so that the buccal surfaces of the teeth were parallel to the table. A treatment window of 4 × 4 mm was exposed on the labial surface of each tooth. Microhardness was assessed with a Vickers hardness apparatus (Matsuzawa, model MHT2, Japan) using a vertical load of 300 g for 10 seconds. The test was performed on three locations at close proximity to each other, and the average Vickers hardness number (VHN) was calculated for each specimen.

The specimens were kept in Fusayama Meyer artificial saliva during the experiment. This solution consisted of KCl (0.4 g/l), NaCl (0.4 g/l), CaCl2.2H2O (0.906 g/l), NaH2PO4.2H2O (0.690 g/l), Na2S.9H2O (0.005 g/l) and urea (1 g/l) with pH=7.03. The artificial saliva was refreshed every day.

-Home bleaching process

**Figure 1:**
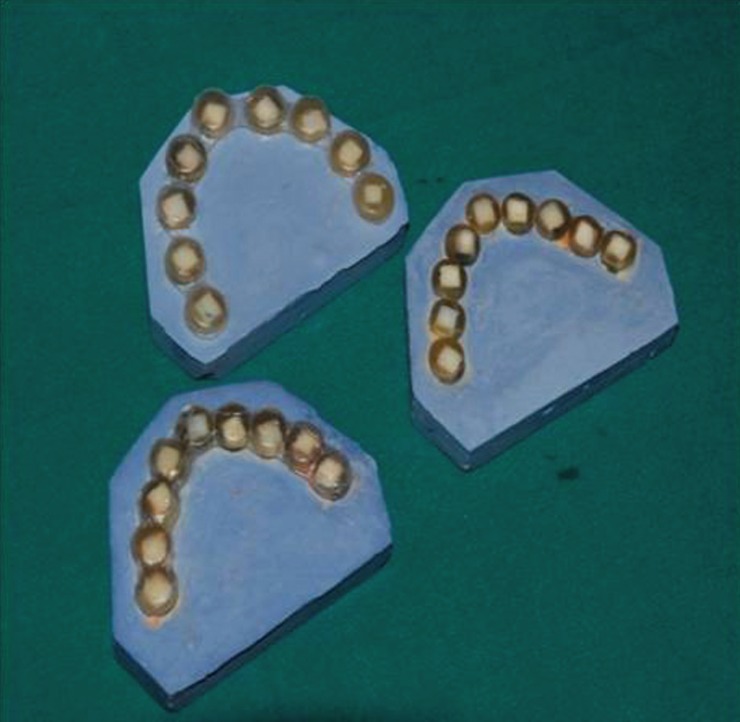
Mounting the specimens for making special trays for home bleaching process.

After measuring the initial VHN, the specimens in the first group underwent home bleaching. For this purpose, the resinous blocks were mounted in plaster so that an ovoid arch was formed from each of the 8-10 specimens (Fig 1), simulating upper dentition. The arch was then covered by two layers of pink wax to allow making a special tray. An alginate impression was then taken and a bleaching tray was fabricated for each dentition.

Before the bleaching procedures, the specimens were dried with cotton rolls to remove artificial saliva. A 15% carbamide peroxide gel (Opalescence; Ultradent Products Inc., South Jordan, UT, USA) was used for home bleaching. The gel was applied on the treatment window of each specimen at an appropriate thickness, approximately 1-2 mm, and then the custom-made tray was placed over the arch. The plaster models were kept in a humid environment. After 8 hours of bleaching treatment, the gel was thoroughly rinsed with water, and the specimens were kept in freshened artificial saliva for the remaining hours of the day. The whitening process was repeated every day over a period of 15 days.

-Laser-assisted in-office bleaching 

In group 2, a 40% hydrogen peroxide gel (Opalescence Xtra Boost; Ultradent Products Inc., South Jordan, UT, USA) was applied on the specimens at an approximate thickness of 1-2 mm, followed by laser irradiation for heat activation of the bleaching gel. The apparatus used was a gallium-aluminum-arsenide (GaAlAs) diode laser (ARC Laser GmbH, Nuremberg, Germany), irradia-ting a wavelength of 810 nm. The laser operated at continuous wave (CW) mode and 2 W of power and was held manually at the closest distance to the specimen using a non-contact handpiece. The in-office bleaching was performed for 3 sessions every seven days, in 15 days. In every session, the bleaching gel was remained on the specimen for a total period of 10 minutes, and the laser was irradiated four times for 15 seconds each at intervals of 2 min between irradiations. The bleaching gel was remained on the tooth for 1 min after the last irradiation, followed by thorough rinsing of the specimen under tap water and storage in artificial saliva.

At the end of the bleaching procedures in the experimental groups, microhardness assessment was repeated under the conditions described previously and at locations close to the previous indentations.

-Statistical analysis

The normal distribution of the data was confirmed by the Kolmogorov-Smirnov test. An independent sample t-test was conducted to compare microhardness of the two groups either before or after the whitening treatment. The change in microhardness of each group after the bleaching process was assessed by a paired sample t-test. The data were analyzed by SPSS (Statistical Package for the Social Sciences, version 16.0, SPSS Inc, IL, USA) software and the significance level was determined at *p*<0.05.

## Results

**Figure 2:**
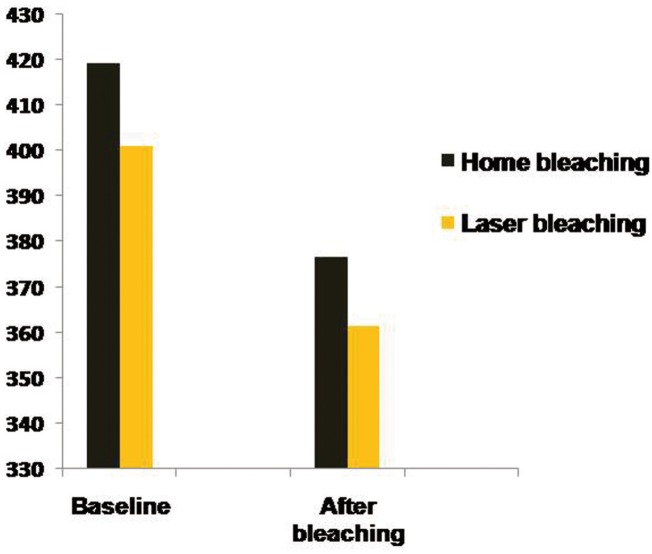
A bar chart comparing enamel hardness in both groups before and after the bleaching procedures.

 Table 1 presents the descriptive statistics including mean and standard deviation (SD) for microhardness of the study groups before and after the bleaching treatments. Microhardness decreased significantly following both home bleaching and laser-assisted in-office bleaching ( Table 1). The percentage reductions of hardness were 10.1% in the first group and 9.9% in the second group. Inter-group comparisons by independent sample t test revealed no significant difference in hardness values among the two groups either before (*p*=0.131) or after (*p*=0.182) the bleaching procedures ( Table 1). Figure 2 compares microhardness of the study groups before and after the bleaching treatments.

**Table 1:**
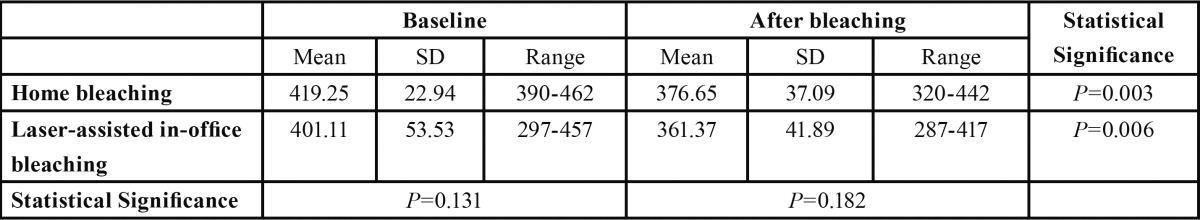
Descriptive statistics and the results of statistical analysis for comparison of microhardness values before and after the bleaching procedures in the study groups.

## Discussion

This study evaluated the effect of two commonly used bleaching techniques including night-guard bleaching with 10% carbamide peroxide and laser-assisted in-office bleaching using 40% hydrogen peroxide on microhardness of demineralized enamel. Hydrogen peroxide, the main component of the bleaching products, produces free radicals after decomposition. These free radicals diffuse into dental structures and oxidize colored molecules, thus resulting in the whitening effect. It has been reported that white spot lesions match better with the remaining tooth structures following the bleaching treatments (8). Microhardness test is a simple and frequently used technique to determine the mechanical properties of enamel and dentine following the bleaching process, and the measurements could be representative for the mineral content of the bleached specimens.

In the present study, both home-bleaching and laser-assisted in-office bleaching resulted in a significant decrease in microhardness of initial caries lesions. The reduction was approximately 10% for the two techniques and indicated a possible alteration in the mineral content of demineralized enamel. The results of this study are in agreement with those of Al-Salehi *et al.* (9), Cavalli *et al.* (13), Borges *et al.* (14) and Globler *et al.* (15) who reported that enamel microhardness significantly decreased upon bleaching. Basting *et al.* (16) also found a significant decrease in microhardness of sound and demineralized enamel submitted to 10% carbamide peroxide bleaching material. Berger *et al.* (10) observed a significant reduction in mineralization level and changes in morphology of dental enamel following bleaching with 35% hydrogen peroxide powered with a halogen light or a LED/diode laser. Some studies indicated more loss of calcium and a decrease in Ca/P ratio in bleached samples (9,17,18). Others observed alterations in enamel surface morphology including pitting, waviness and increased porosity and roughness following bleaching (15,19).

The outcomes of this study, however, are in contrast with some of the previous studies that revealed no significant changes in mechanical, morphological or chemical properties of enamel underwent bleaching treatment with various concentrations of hydrogen peroxide- or carbamide peroxide-containing products (17,20-23). For example, de Magalhaes *et al.* (22) revealed that photoactivation by diode laser did not alter microhardness of human enamel exposed to hydrogen peroxide. Delfino *et al.* (21) noted that microhardness of bovine enamel was not affected by bleaching with 10% or 16% carbamide peroxide gels or a 6.5% hydrogen peroxide strip. Sa *et al.* (23) observed no significant alteration in microhardness of enamel exposed to two in-office bleaching products. The authors (23) assumed that the presence of human saliva could eliminate the demineralizing effects resulted from the low pH of bleaching materials. Other authors (17,24) believed that the changes in microhardness, chemical composition and surface texture of bleached enamel are likely of negligible quantity for clinical aspects. It should be noted that in most of these studies, the healthy enamel surfaces were subjected to bleaching which could exhibit more resistance against the adverse effects of hydrogen peroxide compared to demineralized enamel.

It has been demonstrated that the adverse effects of bleaching products on enamel are directly proportional to the concentration of hydrogen peroxide and duration of bleaching (9,25). Some authors (26) recommended the use of low concentrations of bleaching agents over long periods of time in order to reduce the side effects of bleaching such as tooth sensitivity and gingival irritation. The outcomes of this study suggest that both home-bleaching and laser-assisted in-office bleaching have similar detrimental effects on microhardness of demineralized enamel. Tezel *et al.* (27) also found no significant difference between the calcium loss of the 38% hydrogen peroxide laser-activated group and that of the 10% carbamide peroxide group.

The diode laser was used in this study to accelerate the in-office bleaching process. It is believed that the thermal effect of laser increases the decomposition rate of peroxide and the formation of bleaching active radicals, thus providing the whitening effect in a shorter period of time. The diode laser may also be effective in reducing tooth sensitivity usually observed following the bleaching therapies (28), although there is no clear evidence whether laser application actually decreases or increases the severity of post-operative hypersensitivity. It is obvious that the laser parameters should be kept in a safe range to prevent from temperature elevation in the pulp chamber and thus pulp irritation. We used laser in CW mode at power of 2 W according to the recommendations of the manufacturer. Another option is to use the laser system in pulsed mode which allows sufficient cool down periods between pulses to reduce thermal side effects (29). A special non-contact handpiece was employed for in-office bleaching which expanded the laser beam to include a relatively large area on the specimen and reduce thermal damage to the tooth structure.

In order to inhibit demineralization or manage softened enamel, some studies added additional ingredients to the bleaching products or applied remineralizing agents on bleached enamel (13,14,30). Borges *et al.* (14) reported that the addition of calcium and fluoside into a 35% hydrogen peroxide gel increased the microhardenss of bleached enamel. Cavalli *et al.* (13) reported that mineral loss was minimized by addition of F and Ca to bleaching agents. It should be noted that in the present investigation, the specimens were kept in Fusayama Meyer artificial saliva between the bleaching treatments. This could play an important role in regression of the mineral alterations occurring as a result of bleaching process.

The results of this study suggest that bleaching therapies can aggravate the demineralization process started beforehand and may facilitate the formation of caries cavity in white spot areas, thus compromising long-term tooth health and esthetics. Further studies are warranted to compare the effects of in-office bleaching with different light sources on mineral properties of demineralized enamel. The susceptibility of bleached white spot lesions to the development of caries cavity should also be investigated in the clinical situation. The impact of laser-assisted in office bleaching on intra-pulpal temperature and peroxide diffusion into the pulp also warrants further investigations.

In conclusion, tooth whitening either with home bleaching or with laser-assisted in-office bleaching techniques caused a significant reduction in microhardness of demineralized enamel. Therefore, in patients with white spot lesions, it is suggested to apply remineralizing agents after the bleaching therapy or remineraliz the enamel surface before bleaching.
